# Temporal interpolation alters motion in fMRI scans: Magnitudes and consequences for artifact detection

**DOI:** 10.1371/journal.pone.0182939

**Published:** 2017-09-07

**Authors:** Jonathan D. Power, Mark Plitt, Prantik Kundu, Peter A. Bandettini, Alex Martin

**Affiliations:** 1 National Institute of Mental Health, National Institute of Health, Bethesda, Maryland, United States of America; 2 Department of Radiology, Mount Sinai Hospital, New York City, New York, United States of America; Institute of Psychology, Chinese Academy of Sciences, CHINA

## Abstract

Head motion can be estimated at any point of fMRI image processing. Processing steps involving temporal interpolation (e.g., slice time correction or outlier replacement) often precede motion estimation in the literature. From first principles it can be anticipated that temporal interpolation will alter head motion in a scan. Here we demonstrate this effect and its consequences in five large fMRI datasets. Estimated head motion was reduced by 10–50% or more following temporal interpolation, and reductions were often visible to the naked eye. Such reductions make the data seem to be of improved quality. Such reductions also degrade the sensitivity of analyses aimed at detecting motion-related artifact and can cause a dataset with artifact to falsely appear artifact-free. These reduced motion estimates will be particularly problematic for studies needing estimates of motion in time, such as studies of dynamics. Based on these findings, it is sensible to obtain motion estimates prior to any image processing (regardless of subsequent processing steps and the actual timing of motion correction procedures, which need not be changed). We also find that outlier replacement procedures change signals almost entirely during times of motion and therefore have notable similarities to motion-targeting censoring strategies (which withhold or replace signals entirely during times of motion).

## Introduction

In nearly all fMRI studies, motion is not explicitly measured but is instead estimated from the fMRI data itself. Motion measures are derived from affine transforms produced by rigid body realignment algorithms, usually as one of the first steps in processing a scan. These image-derived realignment estimates, judging from simulated data and optical recordings, are good approximations of the motion that occurs in fMRI data [1–3].

Motion can be estimated at any step during fMRI image processing and is sometimes the first step of image analysis. But in many studies motion estimation is preceded by temporal interpolation of images, either in the form of slice time correction or outlier replacement (e.g., voxel signal despiking). For example, many studies of motion artifact in recent years temporally interpolated images prior to estimating motion [4–10]. Similarly, efforts to find neurobiological bases for motion have used this order of operations [11, 12]. This order of operations is common in the literature: in issues of Neuroimage between September 2015 and January 2016, of 41 fMRI articles that perform slice time correction, 30/41 (73%) performed slice time correction prior to realignment. Relatedly, despiking has been recommended as the first step of image preprocessing by some statistical groups [8].

It matters when motion is estimated in a processing stream because, on first principles, it can be anticipated that temporal interpolation of voxel signals will change brain shape and position. Consider a voxel just beyond the cranium, which is empty space for an entire scan except for a brief head movement into and back out of the voxel (lasting a single volume). If the signal at this voxel is interpolated in time, then, at the time of motion, the post-interpolation value will be created in part from low signal at neighboring timepoints and will therefore be lower than the original value. Symmetric changes will occur in a voxel vacated for the one volume by the brain. The net effect will be to dampen intensity changes that reflect motion, and, in doing so at many voxels, to alter the shape and/or position of the brain. In other words, if temporal interpolation is performed on an fMRI image, it is very likely that less head motion will be in the image afterwards, and that motion estimates will be reduced.

The purpose of this paper is to quantify these predicted effects of temporal interpolation on head motion estimation in fMRI data and to pursue the consequences of reduced motion for later analyses intended to detect motion artifact. We illustrate that motion, and motion artifact, are more easily overlooked using motion estimates derived after temporal interpolation. In other words, a dataset with motion artifact may appear artifact-free if reduced motion estimates are used to find artifact in the dataset. We also find that despiking has a marked tendency to modify images during motion but not at other times, and thus there are important similarities between despiking and motion-censoring processing strategies.

Motion in fMRI images raises a variety of issues related to data quality, including the extent to which the brain is missampled during motion (often called distortion), the extent to which realigned images still exhibit apparent motion (often called misalignment), the ways in which signals are disrupted by motion, and the impact of such disruption on signal covariance (often examined as a function of distance between signals). In the course of creating the data for this paper we generated animations of hundreds of scans that show these phenomena. These animations are available online for immediate viewing or download and may be useful to readers wishing to gain comprehensive viewpoints on data quality and image processing (visit the webpage associated with this article, www.jonathanpower.net/paper-despiking.html, to see the movies, download scripts or fMRI scans, etc.).

## Methods

### Data

Five fMRI cohorts are examined in this report, all of which have been published previously (these are the same data recently examined in [13, 14]). The cohorts will be referred to as the Washington University (WU) cohort, the multi-echo (ME) cohort, the National Institute of Health (NIH) cohort, the Autism Brain Imaging Data Exchange (ABIDE) cohort, and the Brain Genomics Superstruct (GSP) cohort. Each cohort comes from a different physical site and the data as a whole represent a variety of scanner manufacturers and scan sequences. Each subject’s data included a high-resolution T1-weighted (MP-RAGE) scan and one or more resting state T2*-weighted (BOLD-weighted) scans. Field maps were not available for these datasets. Pertinent aspects of the functional data are given here. More details can be found in the references listed. All fMRI datasets were acquired in ascending interleaved order (described here as ‘alt+z’ and ‘alt+z2’, which are AFNI codes for protocols starting on the 1^st^ or 2^nd^ slice, respectively). At the end of each cohort’s description, we list “quality criteria”, which refers to any subject selection procedure related to data quality used to form the cohort by the providers of the data (prior to us obtaining the data).

ME: N = 89 “typical” adults described in [15]; TR = 2470 ms, TE = 12, 28, 44, and 60 ms; voxels = 3.75 x 3.75 x 4 mm; 32 interleaved slices in ‘alt+z2’ acquisition order; Siemens MAGNETOM Trio 3T scanner with a 32-channel receive-only head coil in Cambridge, England; 1 run of 239 volumes (9.8 min) per subject. Although all echoes of the multi-echo data were processed and data from all echoes will be presented, this paper focuses on data from the second echo (TE = 28 ms; this echo time is most similar to that of single-echo sequences). Quality criteria: none that the authors are aware of.

WU: N = 120 “typical” adults described in [16]; TR = 2500 ms; TE = 27 ms; voxels = 4 x 4 x 4 mm; 32 interleaved slices in ‘alt+z2’ acquisition order; Siemens MAGNETOM Trio 3T scanner with a Siemens 12 channel Head Matrix coil in Saint Louis, Missouri, USA; 1 or more runs of varying length across studies: 346 ± 136 volumes are summary mean and standard deviations, representing 14.4 min of data on average. Quality criteria: these 120 datasets were originally chosen out of a larger collection of ~200 datasets at Washington University because they each retain 120+ volumes (~5 minutes of data) even if high-motion volumes are excluded from the scans. This cohort is thus a subsample of the scanned population. These scans are being released with this paper at the website mentioned above.

NIH: N = 91 adolescents and young adults, either typical or high-functioning patients with autism spectrum disorder described in [9]; TR = 3500 ms; TE = 27 ms; voxels = 1.7 mm x 1.7 mm x 3.0 mm; 42 interleaved slices in ‘alt+z’ acquisition order; GE Signa 3T scanner with a GE 8-channel receive-only head coil with SENSE factor of 2 at the NIH Clinical Center NMR Research Facility; 1 run of 140 volumes (8.2 min) per subject. Quality criteria: none.

ABIDE (http://fcon_1000.projects.nitrc.org/indi/abide/): N = 187 children, adolescents, and young adults, either “typical” or high-functioning patients with autism spectrum disorder, representing the CalTech, NYU, UCLA, and USM subgroups of the full ABIDE dataset (these subgroups were chosen because they were already on local servers); all sites used Siemens 3T scanners; the NYU acquisition order was ‘alt+z’ while the others were ‘alt+z2’; Caltech: TR = 2000 ms; TE = 30 ms; voxels = 3.5 x 3.5 x 3.5 mm; 1 run of 150 volumes (5 min); NYU: TR = 2000 ms; TE = 15 ms; voxels = 3 x 3 x 4 mm; 1 run of 180 volumes (6 min); UCLA: TR = 3000 ms; TE = 28 ms; voxels = 3 x 3 x 4 mm; 1 run of 120 volumes (6 min); USM: TR = 2000 ms; TE = 28 ms; voxels = 3.4 x 3.4 x 4 mm; 1 run of 240 volumes (8 min). Quality criteria: none or minimal according to site.

GSP: N = 1000+ “typical” adults described in [17], from which the first 235 subjects were taken (due to limited disk space); TR = 3000 ms; TE = 30 ms; voxels = 3 x 3 x 3 mm; 47 interleaved slices in ‘alt+z’ acquisition order; multiple matched Siemens 3T Tim Trio scanners with Siemens 12-channel head coils in Boston, MA, USA; 2 runs of 124 volumes (6.2 min) per subject. Quality criteria: data were visually screened and pass certain criteria, and are thus a “vetted” set of high-quality scans.

### fMRI data processing: Despiking, slice time correction

This paper focuses on characteristics of fMRI data that 1) have undergone no processing, 2) have been despiked (DS), 3) have been despiked and then slice time corrected (DS+TS), 4) have been slice time corrected (TS), or 5) have been slice time corrected and then despiked (TS+DS). In all datasets, the first 4 volumes were ignored throughout processing to ensure steady state magnetization (except the WU data, which were already at equilibrium by the first acquired volume).

Sometimes images with no processing are presented. These data are often called “Raw” or “Unprocessed”.

Despiking was performed using AFNI’s 3dDespike or SPM’s ArtRepair command; multiple software packages were used to demonstrate that effects are not particular to a specific despiking routine. 3dDespike was used with default settings and the –nomask flag so that the entire volume was despiked. Using the –NEW and –OLD algorithms made no obvious difference except in run time and –NEW results are presented. When ArtRepair (art_despike) was used to despike, settings of Despike = 4 and FiltType = 1 were used, following examples in the script header. These data are often called “Despiked” or “DS”.

Slice time correction was performed with AFNI, SPM, FSL, and the 4dfp tools; multiple software packages were used to demonstrate that effects are not particular to a particular slice time correction routine. The AFNI command “3dTshift” was used, specifying –tzero 0 to shift all signals to the time when the volume began to be collected,–tpattern appropriate to the acquisition sequence (‘alt+z2’ or ‘alt+z’), and specifying the interpolation procedure (–heptic was default, but –linear,–cubic,–quintic, and –Fourier are presented when noted). In other packages: FSL (slicetimer), SPM (spm_slice_timing), and 4dfp tools (interp_4dfp) were used with default settings. These data are often called “Time Shifted” or “TS”.

### fMRI data processing: Realignment

Realignment estimates were derived in AFNI, SPM, FSL, and with 4dfp tools; multiple software packages were used to demonstrate that effects are not particular to a particular realignment routine. The AFNI command “3dvolreg” was used, specifying the maximal iterations (–maxite 25), z-padding 10 (–zpad 10), and the reference volume (the first non-ignored volume of a scan was the default reference, but data using mid-run or run-mean reference volumes, or even volumes from a different scan in the same subject (online movies), are presented when noted). The resampling interpolation was specified using –cubic as the default but using –cubic,–quintic,–heptic, and –Fourier when noted. The FSL command “mcflirt” was used specifying only the reference volume, the SPM command “spm_realign” was used specifying only the reference volume, and the 4dfp command cross_realign_4dfp was used with the appropriate reference volume specified. These settings are thus all essentially “default” settings.

### Data traces

Several traces are used for various analyses in this report.

**Fig 1:**
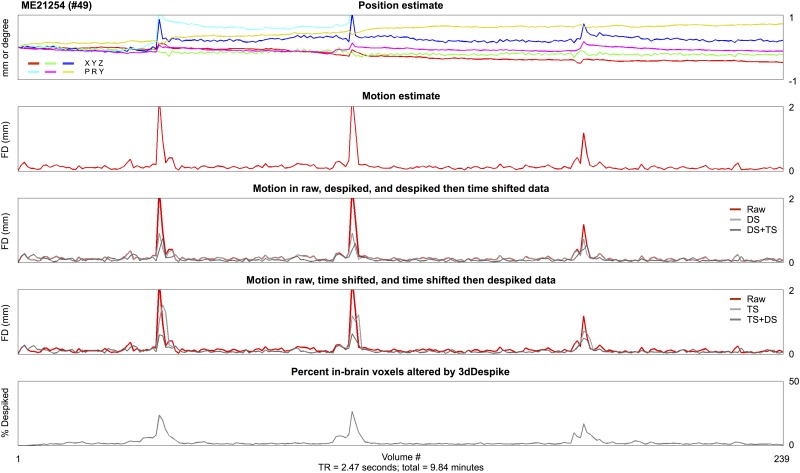
Temporal interpolation reduces motion estimates. All traces are for a single subject of the ME cohort, derived from the second-echo image (TE = 28 ms). At top, position estimates derived from AFNI’s 3dVolreg using heptic interpolation and an early-run reference volume. The corresponding motion (FD) trace is shown immediately below in red, and below that motion derived in the same data after 4 possible orderings of temporal interpolation. At bottom, a trace of the fraction of the brain despiked by 3dDespike (as it operated on raw data to produce the DS data). See S1 Fig for more parameter spaces in the same data (e.g., different TEs, reference volumes, software packages used to estimate motion, etc.). See S2 Fig for the same effects after using other software packages to perform temporal interpolation.

1. Position Estimates: the position of the head as measured by realignment parameters. This report’s positional conventions are RAS: +X +Y and +Z are mm translation in the right, anterior, and superior directions. Rotations are presented in degrees using right-handed conventions: pitch is head nodding with positive = nose up; roll is head tilting with positive = tilt right; yaw is head turning with positive = nose left. Unless otherwise specified, AFNI estimates are shown, but we also present estimates from FSL, SPM, and the 4dfp tools at times.
  Different software packages use different conventions for coordinate origins and for rotational orderings (or quaternions). An implication is that rotational estimates (due to ordering) and translational estimates (due to different compensatory translations for rotations about differing origins) for the exact same correctly-described motion could differ between packages. To minimize such differences, across software packages, we chose outputs using centered conventions when possible. For example, FSL’s mcflirt can output position estimates using corner-of-image conventions (the.mat file) and/or centered conventions (the.par file), and we chose centered conventions. The motion estimates are so similar across packages, even without adjustment for software-specific conventions, that we did not adjust them in Fig 1 and online movies. Readers thus see the similarity between the immediate output of different software packages.
2. Framewise Displacement (FD): framewise displacement indexes change in head position (head motion). FD is calculated as the sum of the absolute values of the differentiated (in time) position estimates, after converting rotational positions to arc length displacements at a radius of 50 mm, as in [4]. By convention, FD = 0 for the first volume.
3. Percent Voxels Despiked: within a subject-specific brain mask, the percentage of in-mask voxels at each timepoint whose signal was changed by 3dDespike (or by ArtRepair if specified).
4. DVARS: an fMRI-image-based measure of how rapidly signal is changing, calculated after [18]: the within-brain root mean square (RMS) value at each timepoint of the differentiated (in time) fMRI image. By convention, DVARS = 0 for the first volume.

### fMRI data co-registration and quality control

To register fMRI data to an atlas space, the subject’s scan-space MP-RAGE was registered using a 12-parameter affine transform to the target atlas (via AFNI’s “@auto_tlrc” command), the first used volume of the first fMRI scan was registered to the scan-space MP-RAGE, and all fMRI scans were registered to the first volume of the first fMRI scan in the motion correction step. These registrations were concatenated to a single transform and the scan-space fMRI data were then transformed to atlas space and resampled to 3 mm isotropic voxels in a single step. The target was the “TT_N27” atlas in AFNI. Note that this procedure enforces cross-run alignment of multiple BOLD runs via the realignment step (note too that realignment estimates produced using the reference volume from the first run were nearly indistinguishable from realignment estimates produced using any run-internal reference volumes (see online movies)).

Each functional scan of each subject was visually checked for goodness of spatial registration and for complete brain coverage in the scan. If coverage or spatial registration was deemed inadequate, the scan (and, if necessary, the subject) was removed from further analysis. This selection based on spatial coverage and registration was the only exclusion criterion for the study.

This exclusion criterion resulted in the loss of 5/89 ME subjects, 3/120 WU subjects, 0/91 NIH subjects, 26/189 ABIDE subjects (3 from UCLA, and all 23 from CalTech; scans in the CalTech site were missing the top of the brain in almost every subject’s fMRI data), and 27/235 GSP subjects.

Readers will note that the critical data in this paper are obtained prior to and independent of registration to a target atlas. We performed registration for two purposes. First, so that realigned data are in a convenient, comparable position across subjects and datasets. Second, so that the data were in register with Connectome Workbench surfaces present in the atlas space (see below).

### Structural data processing

All T1-weighted images in all cohorts underwent automated segmentation by FreeSurfer version 5.3. These segmentations were converted to Connectome Workbench formats as described in [19] to yield 32,492-vertex (“32k”) fs_LR cortical surfaces in both scanner and atlas space for each subject (building on procedures described in [20–25]). These Workbench surfaces are used to sample and present atlas-transformed fMRI volumetric data as described in [19] (building on procedures described in [26, 27]).

### Signals on Workbench surfaces

In the online movies and in some figures, cortical surfaces are used to show the position of the brain and signals at the cortical ribbon. Brains are positioned by the appropriate realignment estimates. For rotations the brain positioning is exact. Vertical and lateral translations on the screen (or page) share gain factors and are nearly exact. In the out-of-screen (or out-of-page) dimension, Workbench does not have a translational parameter but instead an independent “zoom” parameter. This has been set to a gain that is visually congruent with the vertical and lateral displacements. The purpose of these displacements is not analytical and exact, but representative. That surface motions are representative is seen by comparison with the brain motion in companion slices from volumes (in online movies). The signals shown on the surface are generated by sampling the realigned and atlas-transformed volume at the atlas-transformed surface.

### Movies and resources

The 120 datasets from Washington University are being released for public use along with this paper. These data, as well as scripts used to generate the online movies, and other resources, can be found at the website mentioned in the Introduction.

### Analyses

The distance-dependent motion-related analyses follow established conventions reviewed in [28]. For QC:RSFC analyses (QC referring to quality control traces, RSFC referring to resting state functional connectivity measures), mean FD across subjects was the QC vector, and RSFC measures were calculated in each subject as correlations between fMRI timeseries at 264 regions of interest (ROIs) published in [29]. The ROIs were modeled as 10 mm diameter spheres. For each pairwise correlation, the correlation between the QC vector and RSFC measures across subjects was calculated, and this correlation was plotted at the distance between the ROIs yielding the pairwise correlation in question. A smoothing curve over 2000 points was also calculated. All analyses were conducted in fMRI data that have undergone whatever processing is indicated in the figure (Raw, DS, DS+TS, or TS) and that were then transformed into atlas space as described above. The red points in distance-dependent motion plots reflect QC:RSFC correlations in data using the mean FD vector congruent with the data processing. Analyses using other mean FD vectors are summarized using smoothing curves. For scrubbing analyses, the same fMRI data was used as in the QC:RSFC analyses. In these analyses, a threshold (specified in figures, between FD > 0.5 and FD > 0.15 mm) defined volumes to censor, and the difference in correlations in all volumes versus calculated only in volumes under the threshold of motion was calculated and averaged across subjects. Then plots and smoothing curves were created as for the QC:RSFC analyses.

## Results

### Temporal interpolation reduces motion in fMRI images—Single subject evidence

Head position and motion traces are shown in Fig 1 for a single subject’s data. When either despiking or slice time correction routines are applied to the data, motion estimates are reduced, consistent with the reasoning outlined in the Introduction. A variety of factors could in principle also affect motion estimation, such as the reference volume used for realignment or alignment-optimizing cost functions or the software used for realignment. As S1 Fig shows, changing these kinds of parameters produces little to no alteration in estimated motion. S2 Fig demonstrates that the same kinds of motion reductions are caused by despiking and slice time correction routines in AFNI, SPM, FSL, and the 4dfp tools. Thus, no matter the software used to temporally interpolate, or the software used to estimate motion, considerable reductions in motion are caused by temporal interpolation. We show only a single subject here but the effects shown in this subject are characteristic of all subjects; hundreds of similar plots showing similar effects in other subjects can be seen in Online Movies 1 and 2.

**Fig 2:**
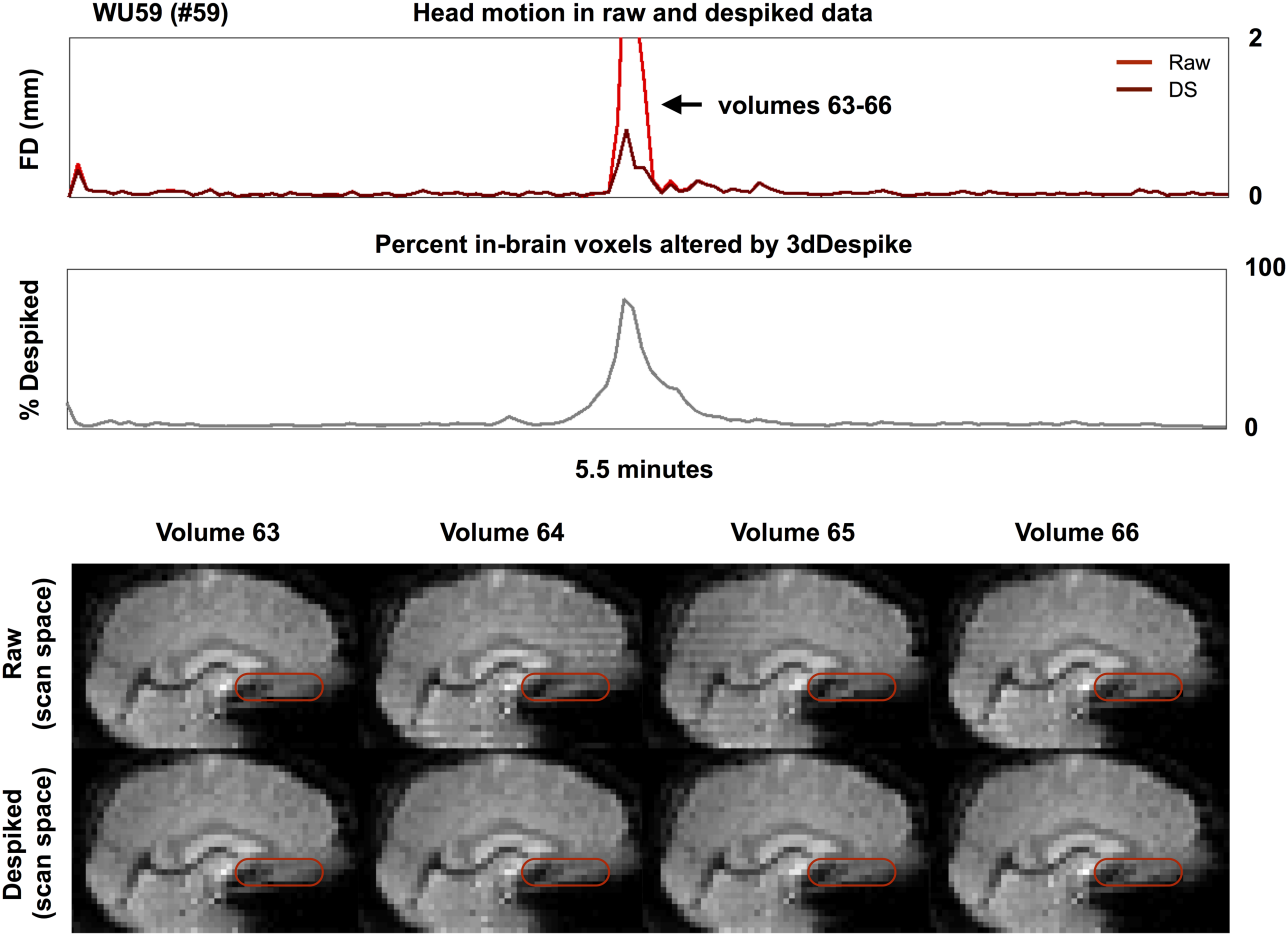
Despiking alters brain position. For a subject of the WU cohort, motion estimates and despike traces are shown at top. Slices of the raw and despiked image are shown spanning a large motion. There is a positive pitch (nose up) rotation at volumes 64 and 65 in the raw data. This motion is attenuated in the despiked data.

The reduced motion estimates reflect the fact that head motion is actually decreased in the scan’s field of view after temporal interpolation. Fig 2 demonstrates one instance of this phenomenon in a single scan. A large head nod (+pitch) at volumes 64 and 65 is visibly reduced after despiking routines—voxels occupied by empty space during the motion have brain tissue in them after despiking (see the red ovals). Another instance of tissue repositioning by despiking, this time of particular slices of a volume, is shown in S3 Fig. Such visibly diminished movements after temporal interpolation are common and can be seen in several hundred stop-animation movies created in single subjects, see Online Movie 3.

### Temporal interpolation reduces motion estimates in fMRI images—Group-level evidence

**Fig 3:**
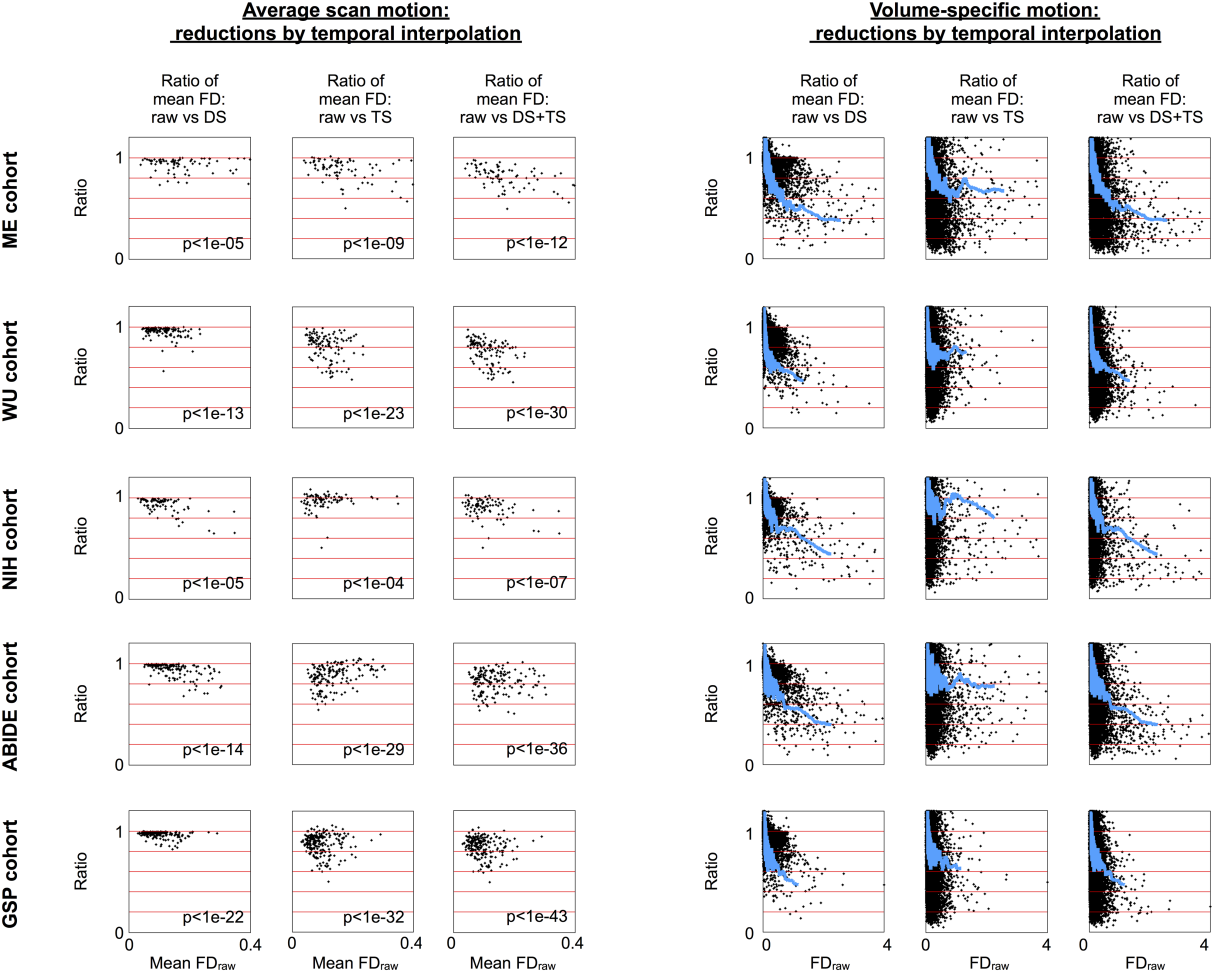
Temporal interpolation tends to reduce estimated brain motion in fMRI scans. Data for 5 cohorts are shown. At left, mean motion in a scan is shown for each subject estimated both before and after temporal interpolation. At right, each volume’s motion is shown in a similar manner with a blue smoothing curve over 2000 points. Raw FD forms the x-axis; the y-axis is the ratio of post-temporal-interpolation motion relative to the raw FD value. Inset p values are from paired t-tests of raw versus post-interpolation mean FD values.

Motion estimates for each subject and each volume of each cohort are shown in Fig 3 for despiked (DS), slice time corrected (TS), and despiked then slice time (DS+TS) corrected data. Both forms of temporal interpolation nearly always reduce mean subject motion, often by 10–30% relative to raw values. S1 Table lists the reductions in mean motion for each dataset across the 4 forms of temporal interpolation: decreases of ~5–10%, ~5–15%, and ~10–20% are seen with despiking, slice time correction, and the combination of techniques, respectively. Both forms of temporal interpolation tend to reduce motion at individual volumes, often by 10–50% or more for volumes with demonstrable motion. These reductions are highly significant. These results confirm that motion estimates are reduced, substantially, and in virtually all subjects, both in individual volumes and at scan-summary levels, when motion is estimated after temporal interpolation versus before interpolation.

### Consequences of temporal interpolation for motion artifact detection and quantification

The changes in head motion described above have several implications for both the presence and detection of motion artifact in fMRI data under different processing strategies. A large literature has emerged in recent years on the detection and quantification of motion artifact, especially in functional connectivity MRI data, reviewed in [28]. Two kinds of analysis will now be used to demonstrate 1) the presence of motion artifact in each of the datasets and 2) how detection of motion artifact can be contingent on the kind of motion estimate and analysis technique being used.

**Fig 4:**
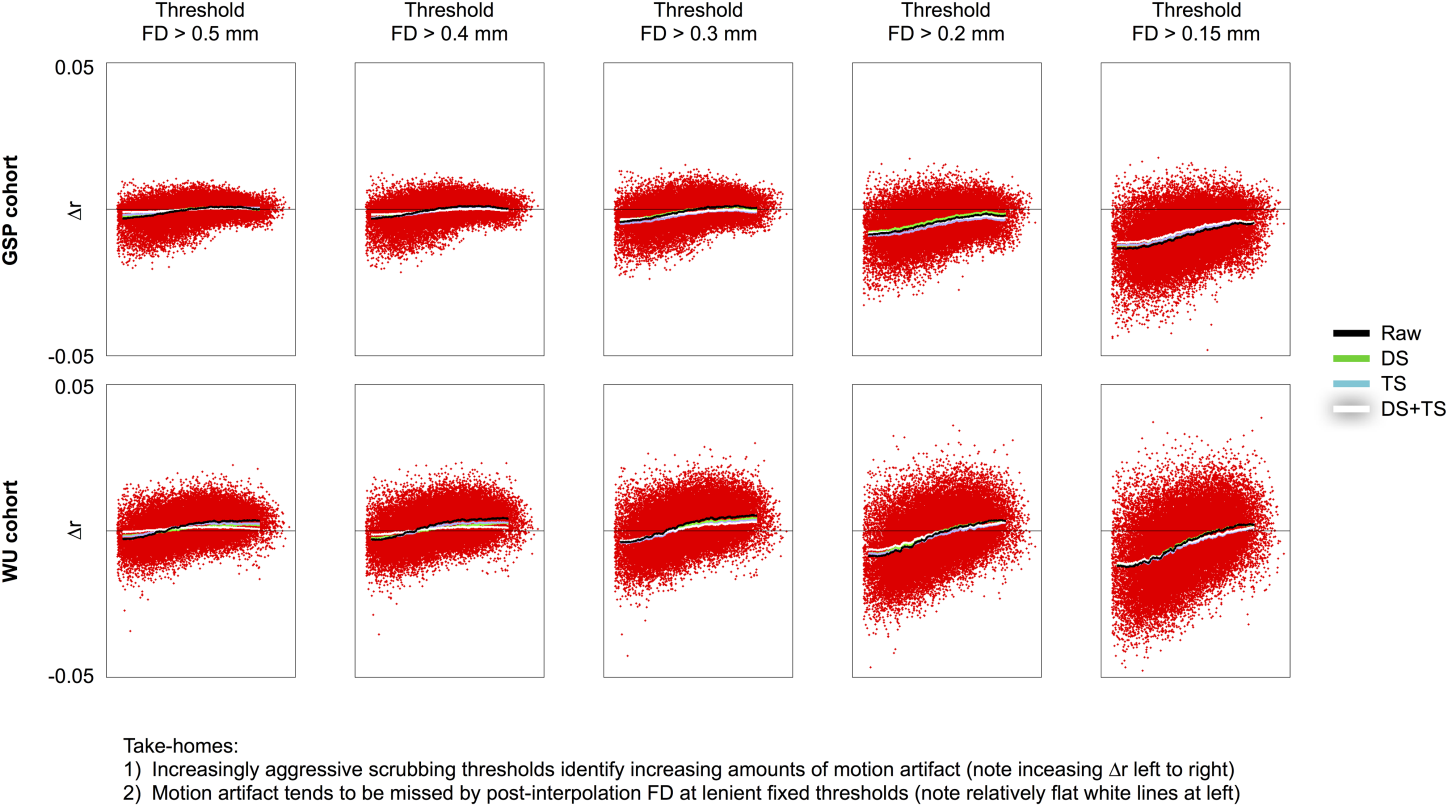
Volumes with motion are most easily identified in raw FD. For the GSP and WU cohorts, scrubbing analyses in raw data are shown using several versions of FD traces at several thresholds. Note that the slope of the red points increases from left to right, indicating that greater amounts of distance-dependent artifact are being characterized at the thresholds approach the “floor values” of the FD trace. Similar data with the same effects are shown for all cohorts in S5 Fig and distributions of each cohort’s FD under various preprocessing schemes are shown in S4 Fig. The x-axis represents distance, spanning 0–180 mm.

One consequence of reduced motion estimates is that they can undermine artifact detection simply because motions do not “stand out” as much after temporal interpolation. That motions are less conspicuous after temporal interpolation is seen in Figs 1 and 2 in the motion traces. That existing motion artifact becomes harder to detect after temporal interpolation will now be demonstrated by scrubbing analyses in all five datasets. Scrubbing analyses are designed to characterize the variance of particular volumes of fMRI scans by showing the difference in correlations produced when certain volumes are withheld from signal correlation calculations. In the following analyses, FD is used to identify volumes to withhold, and FD distributions in each dataset support examination of thresholds down to approximately 0.15 mm (S4 Fig). From left to right in Fig 4, the scrubbing analyses show the effect of using increasingly strict criteria to identify motion, ranging from FD > 0.5 to FD > 0.15 mm. Note that the fMRI data under study here is not changing from left to right, only the data being withheld from correlation calculation changes. The increasing slopes of the resultant curves indicate that increasing distant-dependent artifact is being detected as thresholds drop. This effect is shown for two datasets in Fig 4 and for all datasets in S5 Fig.

An important effect illustrated in Fig 4 is that artifact present in the data can be missed when post-interpolation motion estimates are used to identify motion-related properties: the black trace (raw FD) is sloped at all thresholds, but the white trace (DS+TS) has almost no slope in the left panels. This is because motions that pass high thresholds of 0.5 mm in raw FD are attenuated by temporal interpolation and no longer pass the same threshold. Motion artifact, however, is still present. This “false negative” result can be avoided by using (lower) thresholds that appropriately identify outliers in the FD distribution, see the right sides of Fig 4 or S5 Fig.

**Fig 5:**
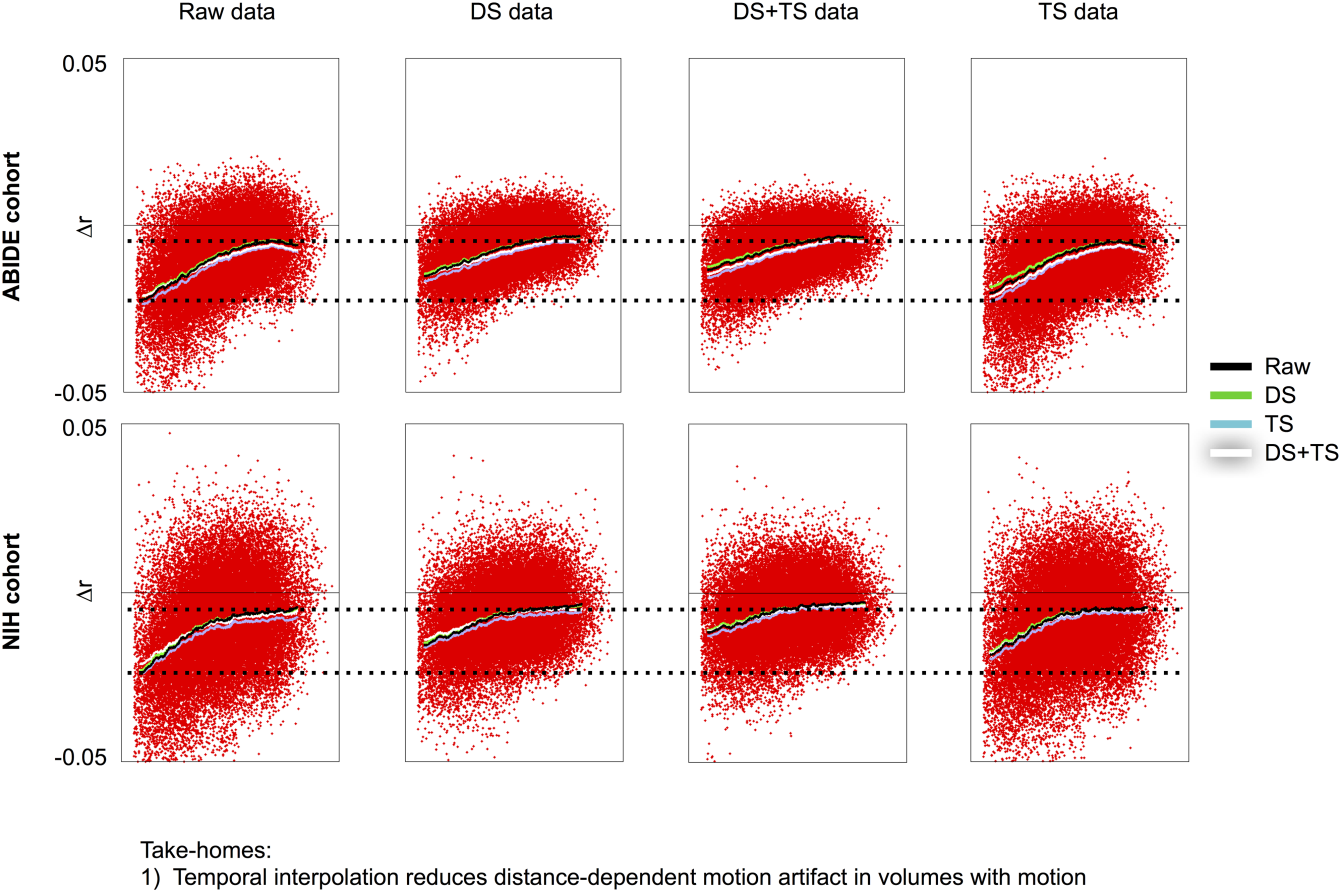
Temporal interpolation reduces distance dependent artifact. For the NIH and ABIDE cohorts, scrubbing analyses in raw, DS, DS+TS, and TS data are shown using several versions of FD traces at a 0.2 mm threshold. From left to right, the volumes being censored are not changing, all that changes is the data undergoing censoring. Note that the slope of the red points decreases in DS and DS+TS data, indicating that smaller amounts of distance-dependent artifact are present in these volumes. Similar data with the same effects are shown for all cohorts in S6 Fig. The same result is seen regardless of threshold used (data not shown). The x-axis represents distance, spanning 0–180 mm.

Another consequence of temporal interpolation is that motion artifact should be somewhat attenuated after interpolation simply because interpolation tends to dampen signal outliers and signal outliers are often artifacts caused by head motion. All five datasets exhibit this effect, shown in Fig 5 and S6 Fig. In this figure, the volumes censored are held constant (defined as those above 0.2 mm for each version of FD) and what changes across the figure is the data that undergo censoring. The salient point is that censoring the exact same volumes produces considerably smaller distant-dependent changes in correlation after the data are temporally interpolated. Stated differently, the slopes of the summary scrubbing curves are shallower in all plots generated in temporally interpolated data, as is the magnitude of changes in correlations, an effect easy to appreciate in the context of the dotted lines placed for reference. This effect is more pronounced in despiked data compared to slice time corrected data.

**Fig 6:**
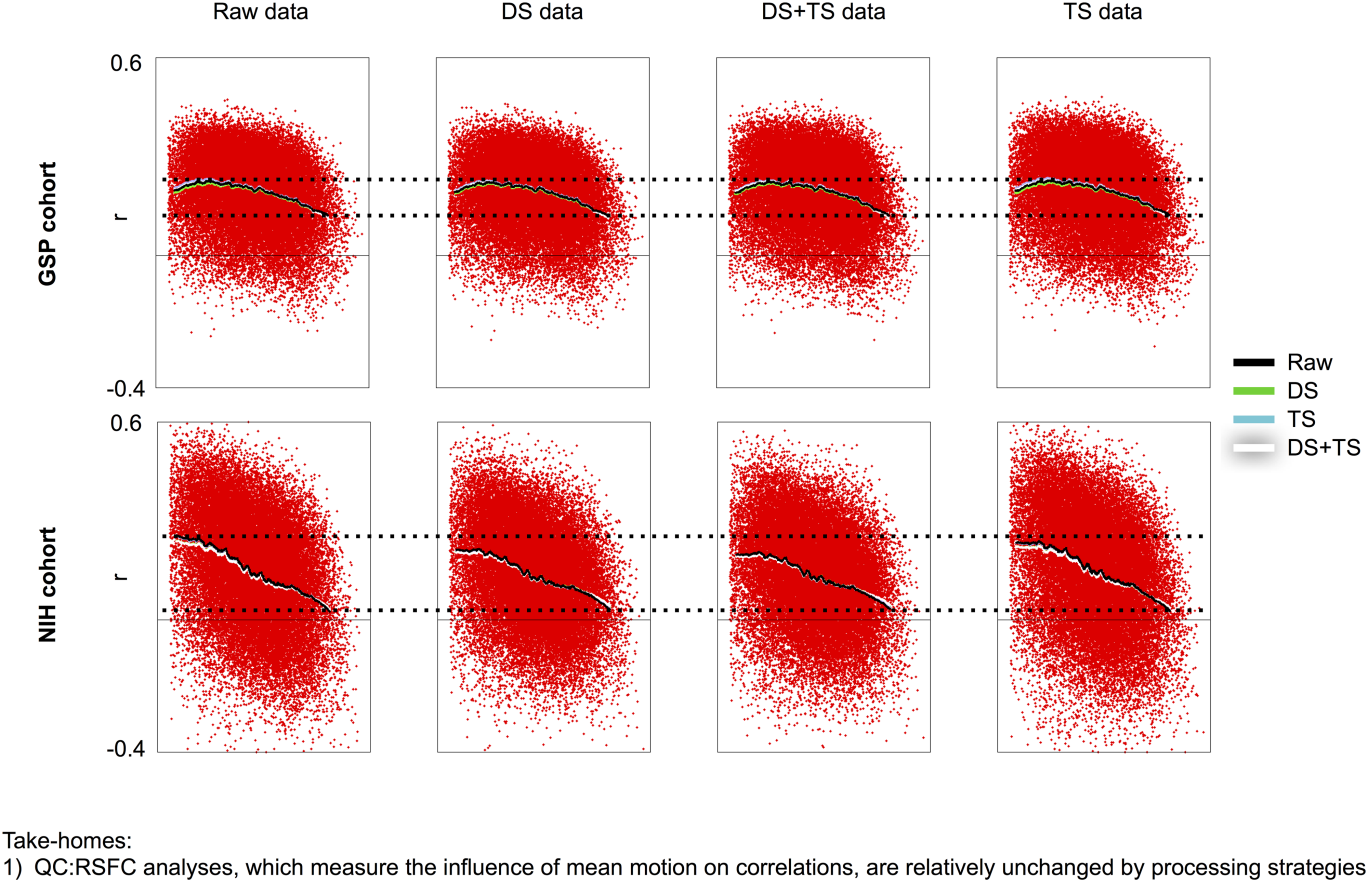
QC:RSFC correlations are not systematically changed across processing strategies. For the GSP and NIH cohorts, QC:RSFC analyses using various FD traces in data from various stages of processing are shown. There is no systematic influence of processing in these cohorts or in the other cohorts (shown in S7 Fig). The x-axis represents distance, spanning 0–180 mm.

Mean scan motion can also be used to characterize motion-associated variance via QC:RSFC analyses (reviewed in [28]). In the following analyses, instead of examining how correlations change when certain volumes are withheld from calculations (scrubbing analyses), we now examine how correlations are modulated by the mean motion in each scan, and we vary both data processing and the kind of FD trace used to form the mean FD vector. Fig 6 shows such analyses for 2 cohorts (all cohorts show the same effect and are shown in S7 Fig). Distance-dependent motion artifact is present in all cohorts, under all processing strategies, and the slope of the distance dependence is not systematically changed across mean FD vectors. Although there is some attenuation of the slope in temporally interpolated data, the effect is slight and not as pronounced as in the scrubbing analyses.

## Discussion

The central concepts in this paper are 1) outliers in voxel signals prior to realignment can reflect changes in brain position, 2) temporal interpolation of voxel signals prior to realignment will tend to reduce motion in the field of view and thus estimated motion, and 3) reduced motion estimates make it harder to recognize the times at which motion artifact occurs in datasets. This latter point may be especially relevant to studies of dynamics in fMRI timeseries, in which it is important to recognize and account for varying amounts of motion across time [30].

### Basic expectations of motion artifact in fMRI data

This study examined five large fMRI datasets acquired at different sites by different investigators, each consisting of ~90–230 subjects. In each dataset, distance-dependent motion artifact was present and revealed by both scrubbing and QC:RSFC analyses (Figs 4–6). Importantly, in each of these datasets, distance-dependent artifact is present specifically in the volumes during which motion is occurring, demonstrated by the scrubbing analyses. These effects resemble effects in other published datasets (e.g., [6, 31]). Collectively these results indicate that a wide variety of fMRI datasets share the following basic characteristics: short-distance correlations are increased relative to long distance correlations in datasets with more motion, and correlations at all distances tend to be increased in datasets with more motion.

If a dataset is reported to not exhibit motion artifact, it is sensible to examine carefully the evidence on which such a claim is based. Three potential ways that “false negatives” can occur in motion artifact detecting analyses will be mentioned here. First, one way for a “false negative” to occur is to have a too-high threshold defining volumes to censor. For example, in Fig 4, after temporal interpolation, motion had been reduced to an extent that almost no motion artifact was detected at thresholds that yielded motion artifact prior to temporal interpolation. Note that artifact remained in these scans after temporal interpolation, it just became harder to detect. A second kind of “false negative” can occur in QC:RSFC analyses when data from multiple sites with substantially different cross-site signal properties are combined such that systematic within-site mean-FD-dependent properties are obscured by systematic cross-site differences. We have observed this phenomenon in multi-site clinical data not presented here. A third way in which motion artifact can be overlooked is if a handful of outlying datasets are allowed to influence the scrubbing or QC:RSFC correlations calculated across many subjects; correlations are sensitive to outlying values and a few scans with marked abnormalities can obscure relationships present across most other datasets.

### On interpreting published motion estimates and motion-related analyses

Mean motion is often reported as a proxy for data quality. In this report, measures of mean FD were usually reduced by ~5–10% by despiking, by ~5–15% by slice time correction, and by ~10–20% when these steps were combined. Motion estimated after temporal interpolation can thus yield more favorable “quality” estimates, a fact useful to keep in mind when comparing studies.

In this report, estimated motion in individual volumes is often decreased by 10–50% or more by despiking and/or slice time correction. Large movements are often greatly reduced and may appear innocuous, and modest motions that have consequences in signals may no longer stand out sufficiently to be easily identified. Much of the recent literature on motion artifact performed despiking and/or slice time correction prior to realignment (e.g., [4–10, 32]). It thus seems likely that motion artifact was somewhat under-identified in these reports.

Multiple techniques can be used to investigate the consequences of motion, but they do not capture the same phenomena. Visual inspection of FD traces indicated decreased motion after temporal interpolation in virtually all subjects (Figs 1–3). Scrubbing analyses indicated that the amount of detected distance-dependent artifact was decreased in motion-containing volumes both when post-interpolation motion estimates were used to find motion (Fig 4) and in data that had undergone interpolation (Fig 5). However, there was little change across processing strategies or FD traces in QC:RSFC analyses (Fig 6). The discrepancy between artifact detected by scrubbing and QC:RSFC analyses arises because the largest signal changes occur at times of motion and thus the effects of temporal interpolation are most dramatic at times of motion. Analyses targeting these timepoints (e.g., scrubbing analyses) will be maximally sensitive to such time-specific effects, which are “diluted” across time in the QC:RSFC analyses. A corollary of this point is that analyses targeting specific points in time, for example studies of “dynamics”, will be especially susceptible to overlooked (i.e., unrecognized) instances of motion (see, e.g., [30]). Future analyses may extend these observations to other datasets (e.g., the Human Connectome Project data), to other outcome measures, and other atlases.

### A comment on the composition of volumes acquired during motion

**Fig 7:**
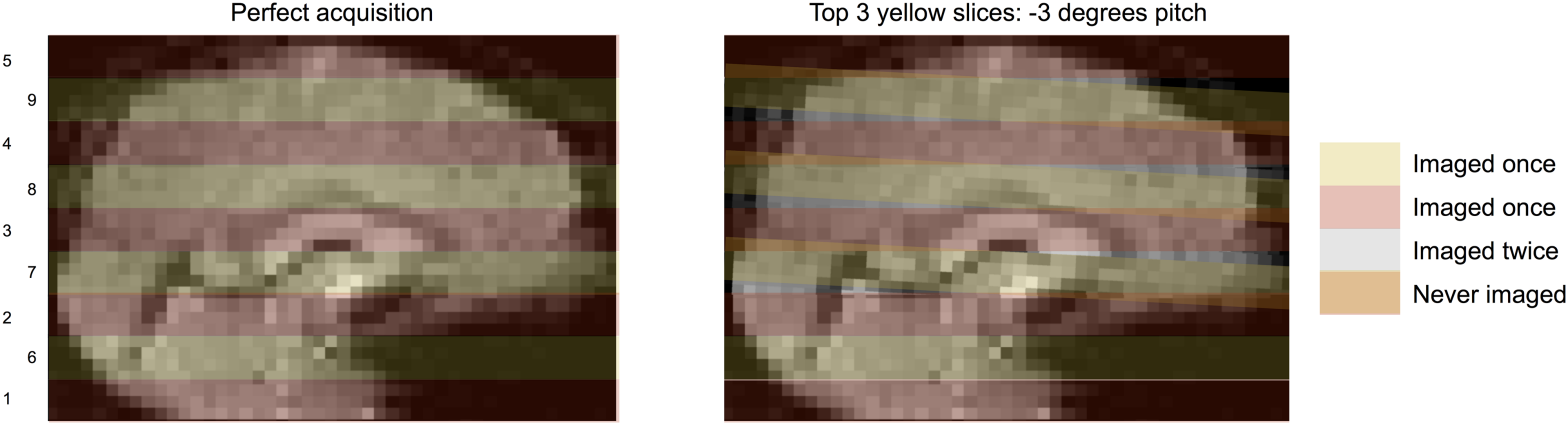
The meaning of image “distortion”. Consider an image acquired in interleaved, ascending fashion. When motion occurs (here, affecting slice acquisitions 7, 8, and 9), the shape of the brain is altered. This shape is often referred to as distorted, but distorted does not mean “warped”. Rather, distortion means that certain parts of the brain are never scanned during that timepoint, and other parts of the brain are scanned twice. There is no way to recover the missing information.

Volumes acquired during motion are fundamentally incomplete samplings of the brain that prevent proper alignment of brain tissue. Any motion with a through-plane component (virtually all motions) implies that some brain tissue will not be scanned in that volume (because the tissue moves from a to-be-scanned slice into an already-scanned slice), and that some tissue will be scanned multiple times (by moving from an already-scanned slice into a to-be-scanned slice). Thus, when images are distorted by anything other than within-slice movement, the distortion entails partially incomplete and partially duplicated samplings of brain tissue (illustrated conceptually in Fig 7). In other words, “distortion” of the brain is not distortion in the sense of warping (like a fun house mirror), but in the sense of an incompletely and incorrectly sampled brain (more like a shattered mirror with missing and displaced pieces). It is more difficult for an investigator to recognize when such missampled volumes are acquired if motion is estimated following temporal interpolation of the image.

### A comment on the uncertainty of tissue “alignment” after temporal interpolation

Consider the following illustrative scenario: a brain entirely still during a scan except for a single transient motion producing displacement in a single slice. For simplicity let us presume this conceptual motion is entirely in-plane, and that the data are despiked and then realigned.

Non-despiked tissue may be misaligned via the following despiking-related mechanism. A signal must meet certain criteria of signal change to be despiked. Voxels near the boundaries of tissue types with differing intensity are most likely to display large signal changes during motion, and are thus the most likely candidates for despiking. We will call these Type 1 voxels. Now consider voxels not on boundaries, such as adjacent voxels in gray matter. If tissue shifts in-plane through these voxels, gray matter replaces gray matter in a voxel and signal intensity is not likely to change as greatly. These voxels are less likely to be despiked. We will call these Type 2 voxels.

Let us say that all voxels in the conceptual slice in question experienced translation Δy, but mostly voxels on the boundaries of different tissue types (Type 1) met criteria for despiking. Motion estimates would thus be reduced below Δy (by cropping the “jutting out” and “jutting in” portions at the edge of the brain as discussed in the Introduction, see S3 Fig for a real example), but non-despiked voxels (Type 2) contain tissue still shifted Δy, so that when realignment is applied, the non-despiked Type 2 voxel signals are not quite corrected in their position, since estimated motion will be below Δy. In this way, non-despiked Type 2 signals can be spatially misplaced due to despiking-related changes in motion estimates.

This scenario can be relaxed to include any kind of motion. The phenomenon is only contingent on some kinds of voxels being more changed than other voxels by a temporal interpolation. The extent to which this kind of tissue misplacement occurs and/or matters is unknown and will likely depend on characteristics of the data such as voxel size, the kind and amount of motion, and the kind of temporal interpolation applied to the data. Such considerations reinforce the ambiguities of signal “goodness” during motion-containing volumes.

### On measuring and correcting different kinds of motion in the brain

Motion in fMRI images has many forms. Within a completely still cranium, the brain pulsates by 50–100 μm during systole [33]. This scale of motion was relatively small when voxels were 3–4 mm on a side but becomes of increasing relevance as voxel sizes now approach 1 mm in multi-band sequences. Further, the head continuously and rhythmically bobs with heartbeat and respiration, a phenomenon that becomes more apparent in fMRI motion estimates as TRs are reduced to intervals capable of sampling these phenomena (e.g., respiratory motion becomes readily apparent once TRs drop to 1000 ms or less in multiband sequences, [31]). And further, there are intermittent and often rather large motions that are easily appreciated in FD traces or even by eye. The measurement, consequences, and correction of the first two kinds of motion are not well-studied presently, in part because the spatial and temporal scales of these phenomena were not practically accessible or relevant prior to a few years ago. The latter kind of motions, which are larger and more sporadic, have been the subject of much work in recent years.

An incidental finding in this paper, seen most easily in Online Videos 1 and 2, is that the motion estimates returned by a variety of software packages and under a variety of processing decisions (excluding temporal interpolation) are very similar and reliably identify the same large, sporadic movements in a scan. For example, correlations of FD traces for a scan across software packages (AFNI, FSL, SPM, and 4dfp tools) are r = 0.95 ± 0.05 across subjects. Similarly, correlations of FD traces for a scan when using different volumes as reference images (e.g., mid-scan versus image mean) are near r = 1 across subjects. And separate estimation of motion at different TEs in multi-echo scans yield estimates that are nearly identical across TEs. Investigators may thus have confidence that image-derived motion estimates capture similar large-scale motions under most circumstances.

Some readers may wonder whether one or another version of the motion estimates (e.g., estimates in unprocessed data versus temporally interpolated data) most closely resembles the “ground truth” movement. Our data cannot speak directly to this issue since we lack external measures of motion. It is certain that in general for any “real” amount of motion, temporal interpolation will reduce that motion. It seems more important, however, to emphasize that there is not always a “ground truth” to an fMRI motion estimate since it is a point estimate of a continuous process. For example, if a brain is unmoved partially through a volume acquisition and then moves and the volume acquisition finishes, a rigid body realignment estimate for the volume cannot possibly capture the fact that some of the brain is still and some is moved. The “ground truth” is that each slice, to the extent that it is nearly instantaneously acquired, contains a certain slab of brain tissue that has moved a certain amount from the last time the slab was acquired, and this motion may be different from the motions that describe other slabs and/or slices of the volume.

In summary, the brain is in constant motion for a variety of reasons at a variety of spatial scales. With regard to large motions of the entire head, simulations and optical recordings indicate that fMRI motion estimation procedures are reasonably accurate [1–3], and our results above indicate that motion estimates are fairly reliable across software packages and parameter spaces. On the other hand, the presumptions of rigid body alignment are routinely violated and it is clear that single point estimates cannot capture the full timing and trajectories of the motions the head describes during a TR. Motion estimates are approximations.

The considerations outlined in the last three sections illustrate the challenges posed by motion for data quality and tissue alignment: the brain is missampled and it is difficult to put signals in exactly the right place with confidence during such volumes. Slice-specific motion correction does not avoid these challenges because the fundamental issue is that some tissue has, improperly, not been sampled, whereas other tissue, again improperly, has been sampled multiple times during a TR. Prospective correction during acquisition could in principle correctly sample the brain and enable proper registration and alignment during motion [34, 35]. These techniques are under active development for BOLD-weighted sequences but they are not yet widely used and they do not yet avoid abnormalities in volumes acquired during motion.

### On similarities between despiking and motion censoring approaches

Our results indicate that despiking mostly occurs during motion. Additionally, despiking can “bleed out” from times of motion because some window of data must be used to define “normal” and “abnormal” variance (e.g., note the shoulders of the despiking trace in Fig 2, which both precedes and follows the period of motion). The correlation between FD traces and despiking traces in each dataset are: ME: 0.69 ± 0.26; WU: 0.66 ± 0.28; NIH: 0.67 ± 0.34; ABIDE: 0.65 ± 0.36; GSP: 0.56 ± 0.28, all highly significant correlations. Given that despiking procedures replace substantial portions of many motion-exhibiting volumes (often 20–80% of voxels) with data derived from surrounding timepoints, investigators should consider whether such heavily-despiked timepoints ought to be statistically treated the same as lightly-despiked timepoints, and the extent to which such treatment is distinct from or desirable to fully replacing the volume by interpolation or entirely censoring the volume. Stated plainly, if investigators are concerned about censoring volumes that show evidence of motion, they ought also to be concerned about despiking scans, since despiking targets instances of motion and replaces the signals at those times with signals derived from other timepoints. The statistical implications of such procedures, in terms of degrees of freedom, will naturally depend on the signal frequencies under study, the kinds of processing applied to the data, and the temporal clustering of volumes undergoing censoring or replacement (see the discussion on interpolation and censoring in [28]). A full treatment of these issues is beyond the scope of this article.

### Considerations about data processing

Slice time correction is a well-motivated processing step [36]. Despiking can also be well-motivated. The findings of this paper should not be construed as arguments against either of these procedures, but as warnings that motion can be greatly attenuated following such steps. Our findings suggest that it would be useful to run realignment algorithms on data prior to temporal interpolation, simply to obtain motion estimates. Such “raw” estimates will be independent of any subsequent processing stream, have higher sensitivity for detecting motion and motion artifact, and will be more comparable across studies compared to estimates obtained after temporal interpolation. Obtaining “raw” estimates does not preclude performing image realignment at any other point of a processing stream.

## Supporting information

S1 Fig Despiking and slice time correction reduce motion estimates. For a subject of the ME cohort, several traces are shown. At top left, position estimates in the raw second-echo images (TE = 28 ms) are shown, derived from AFNI’s 3dVolreg using heptic interpolation and an early-run reference volume. The corresponding motion (FD) trace is shown immediately below in red. The DVARS trace for the image is shown in blue. A trace of the percent of in-brain voxels despiked by 3dDespike is shown in gray. All other panels show motion (FD) traces when various parameters are altered, including echo time, reference volume, interpolation technique, software package used to calculate position estimates, and various processing steps that can precede image realignment. S2 Fig shows similar traces using alternate software implementations of despiking and slice time correction.
(TIFF)

S2 Fig Decreases in motion estimates are produced by slice time correction and/or despiking in several major software packages. Plots follow conventions of Fig 1 and S1 Fig. Default settings are used for slice time correction in AFNI, FSL, SPM, and 4dfp tools. Despiking is performed in AFNI and SPM, using default settings.
(TIFF)

S3 Fig Despiking alters brain shapes. For a subject of the ME cohort, FD traces in raw and despiked data are shown at top, as is a trace of the percent voxels despiked by 3dDespike. The grayscale panel shows the timeseries of 264 regions of interest from (Power et al., 2011) that span much of the cortex, subcortical nuclei, and cerebellum. Below that, to convey the action of 3dDespike, 3 randomly chosen in-brain voxel signals are shown before and after despiking (signals are vertically offset on the y-axis to ease visualization; ticks represent 5% signal). At bottom, slices of volumes before, during, and after a motion are shown. In raw data, the brain is displaced in several slices, distorting the brain’s shape (red arrows). In despiked data, the shape of the brain during motion has been altered to be smooth like the preceding and following volumes.
(TIFF)

S4 Fig FD distributions under several processing regimes. Data from each cohort is shown in a row. At left, the distribution of FD values. At middle, the percent of volumes under censoring thresholds. Values in the tables at right are drawn from the middle plots. Raw shows the percent of volumes under the threshold in raw data, and for other processing regimes, the percent additional (+) or less (-) volumes under the threshold is shown.
(TIFF)

S5 Fig Volumes with motion are most easily identified in raw FD. As for Fig 4 but with all cohorts. At far left and far right, rather than using a fixed threshold for all data, the percent of volumes in each subject censored by raw FD is used to define (lower) thresholds for the other post-temporal-interpolation versions of the FD traces so that all FD traces censor the same fraction of volumes. At more lenient thresholds, this has the effect of rendering post-interpolation FD traces more sensitive to motion (note that white curves better approximate the black curves). At stringent thresholds, post-slice-time correction curves (blue and white) are systematically shifted down, suggesting that they are shifted in time such that they increasingly identify a global signal that elevates correlations throughout the brain (hence the pan-distance decreases in correlations upon censoring). The x-axis represents distance, spanning 0–180 mm.
(TIFF)

S6 Fig Motion artifact is reduced in despiked data. As for Fig 5, but with all cohorts. The x-axis represents distance, spanning 0–180 mm.
(TIFF)

S7 Fig QC:RSFC correlations are not systematically changed across processing strategies. As in Fig 6, but with all cohorts. The x-axis represents distance, spanning 0–180 mm.
(TIFF)

S1 Table Mean and standard deviations in each dataset of the mean motion of all subjects. DS means despiked, TS means slice time corrected.
(DOCX)
