# Supplementary figures and images for: Temporal interpolation alters motion in fMRI scans: Magnitudes and consequences for artifact detection
Source: PLoS One. 2017 Sep 7;12(9):e0182939. doi: 10.1371/journal.pone.0182939 (PMC5589107; doi:10.1371/journal.pone.0182939)

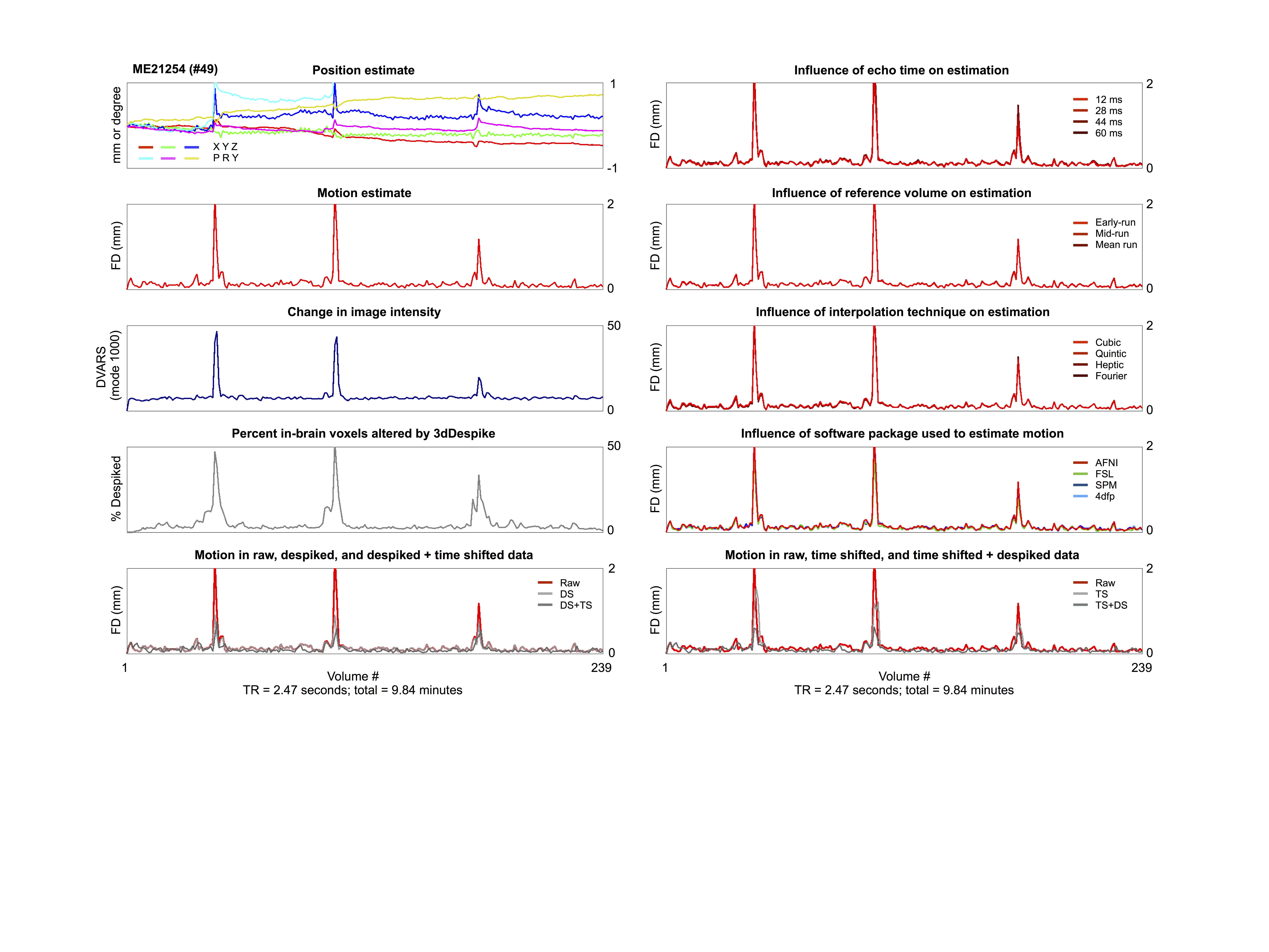

Supplement: S1 Fig — For a subject of the ME cohort, several traces are shown. At top left, position estimates in the raw second-echo images (TE = 28 ms) are shown, derived from AFNI’s 3dVolreg using heptic interpolation and an early-run reference volume. The corresponding motion (FD) trace is shown immediately below in red. The DVARS trace for the image is shown in blue. A trace of the percent of in-brain voxels despiked by 3dDespike is shown in gray. All other panels show motion (FD) traces when various parameters are altered, including echo time, reference volume, interpolation technique, software package used to calculate position estimates, and various processing steps that can precede image realignment. S2 Fig shows similar traces using alternate software implementations of despiking and slice time correction. (TIFF) [file pone.0182939.s001.tiff]

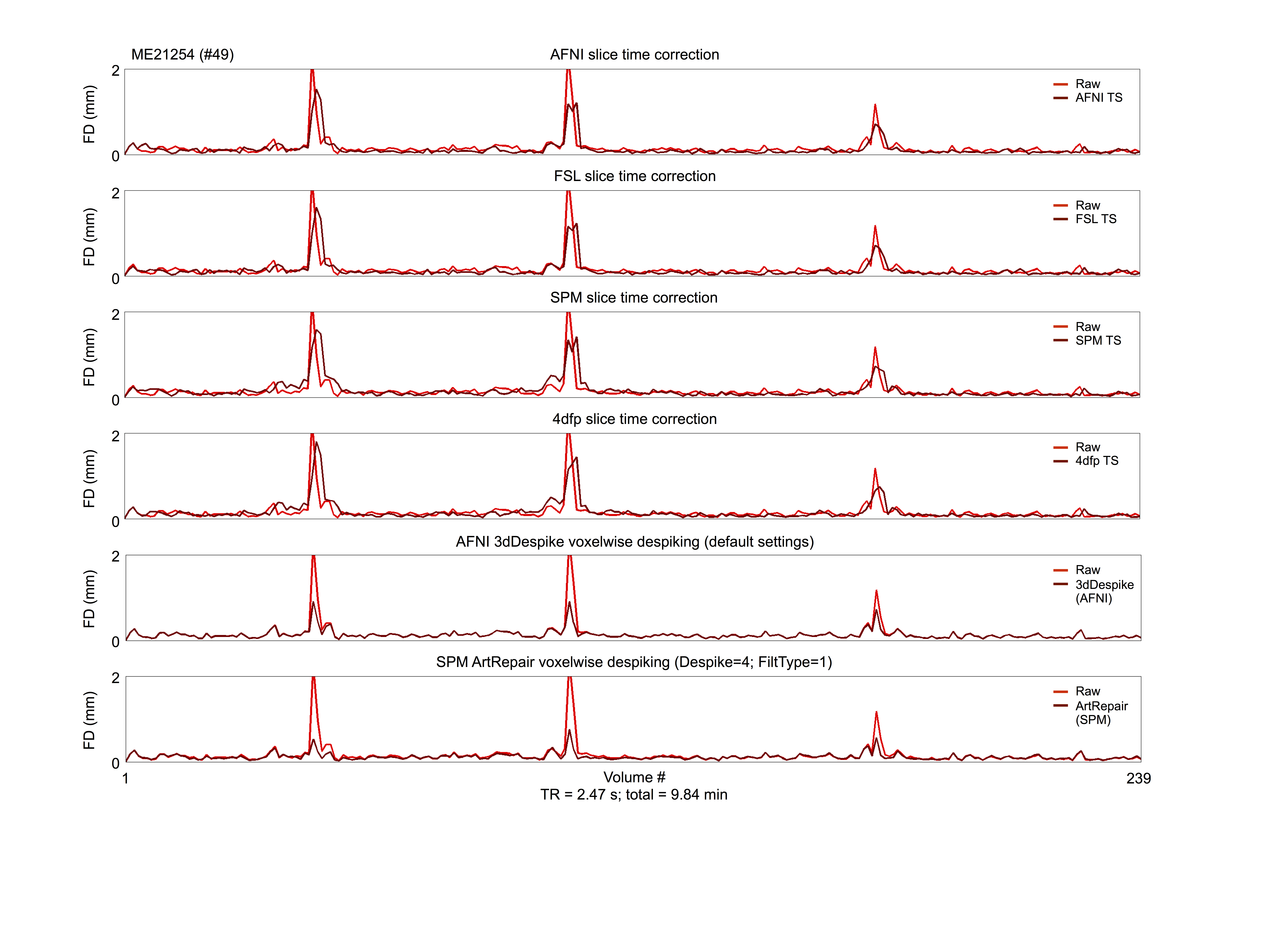

Supplement: S2 Fig — Plots follow conventions of Fig 1 and S1 Fig. Default settings are used for slice time correction in AFNI, FSL, SPM, and 4dfp tools. Despiking is performed in AFNI and SPM, using default settings. (TIFF) [file pone.0182939.s002.tiff]

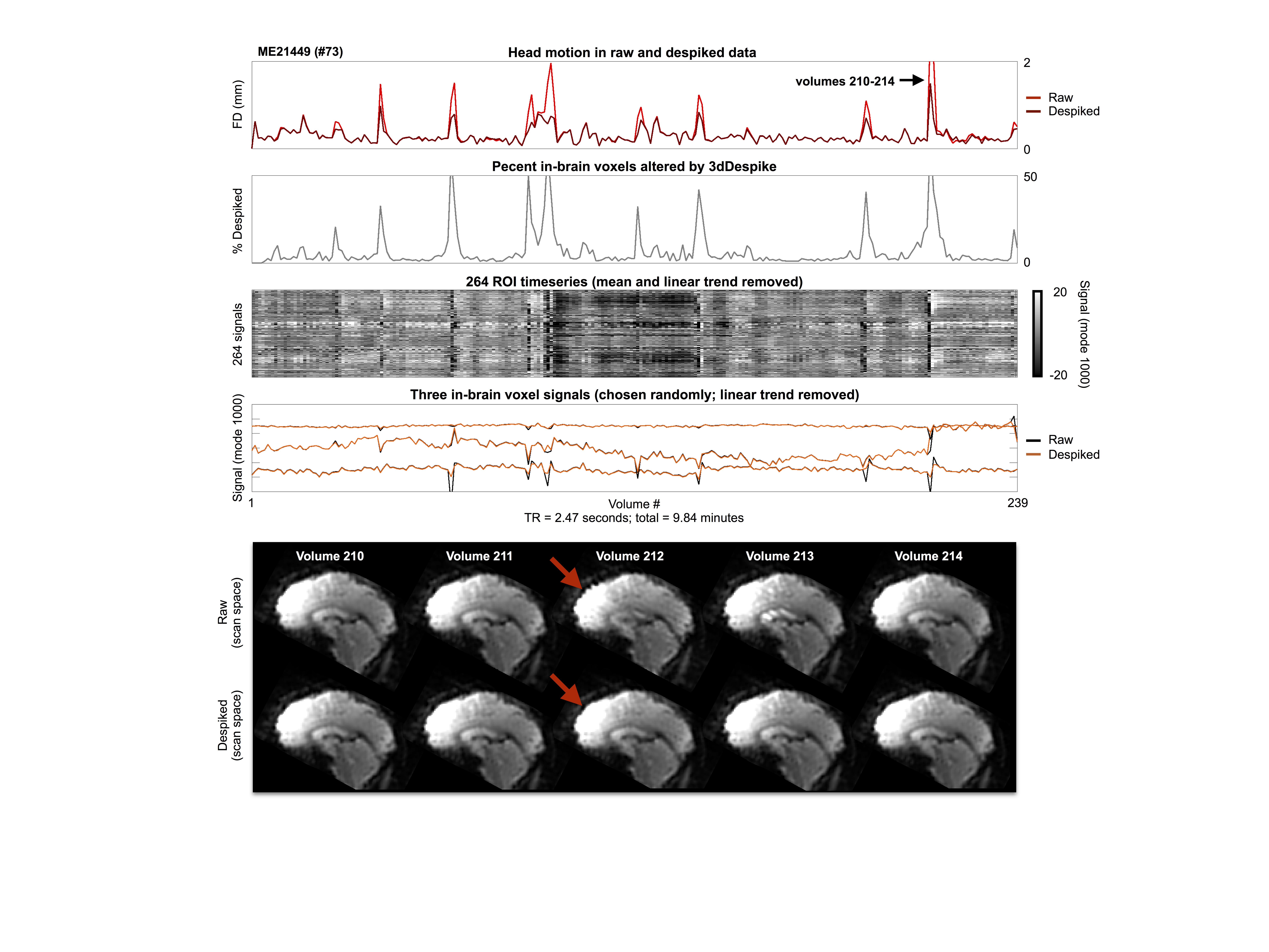

Supplement: S3 Fig — For a subject of the ME cohort, FD traces in raw and despiked data are shown at top, as is a trace of the percent voxels despiked by 3dDespike. The grayscale panel shows the timeseries of 264 regions of interest from (Power et al., 2011) that span much of the cortex, subcortical nuclei, and cerebellum. Below that, to convey the action of 3dDespike, 3 randomly chosen in-brain voxel signals are shown before and after despiking (signals are vertically offset on the y-axis to ease visualization; ticks represent 5% signal). At bottom, slices of volumes before, during, and after a motion are shown. In raw data, the brain is displaced in several slices, distorting the brain’s shape (red arrows). In despiked data, the shape of the brain during motion has been altered to be smooth like the preceding and following volumes. (TIFF) [file pone.0182939.s003.tiff]

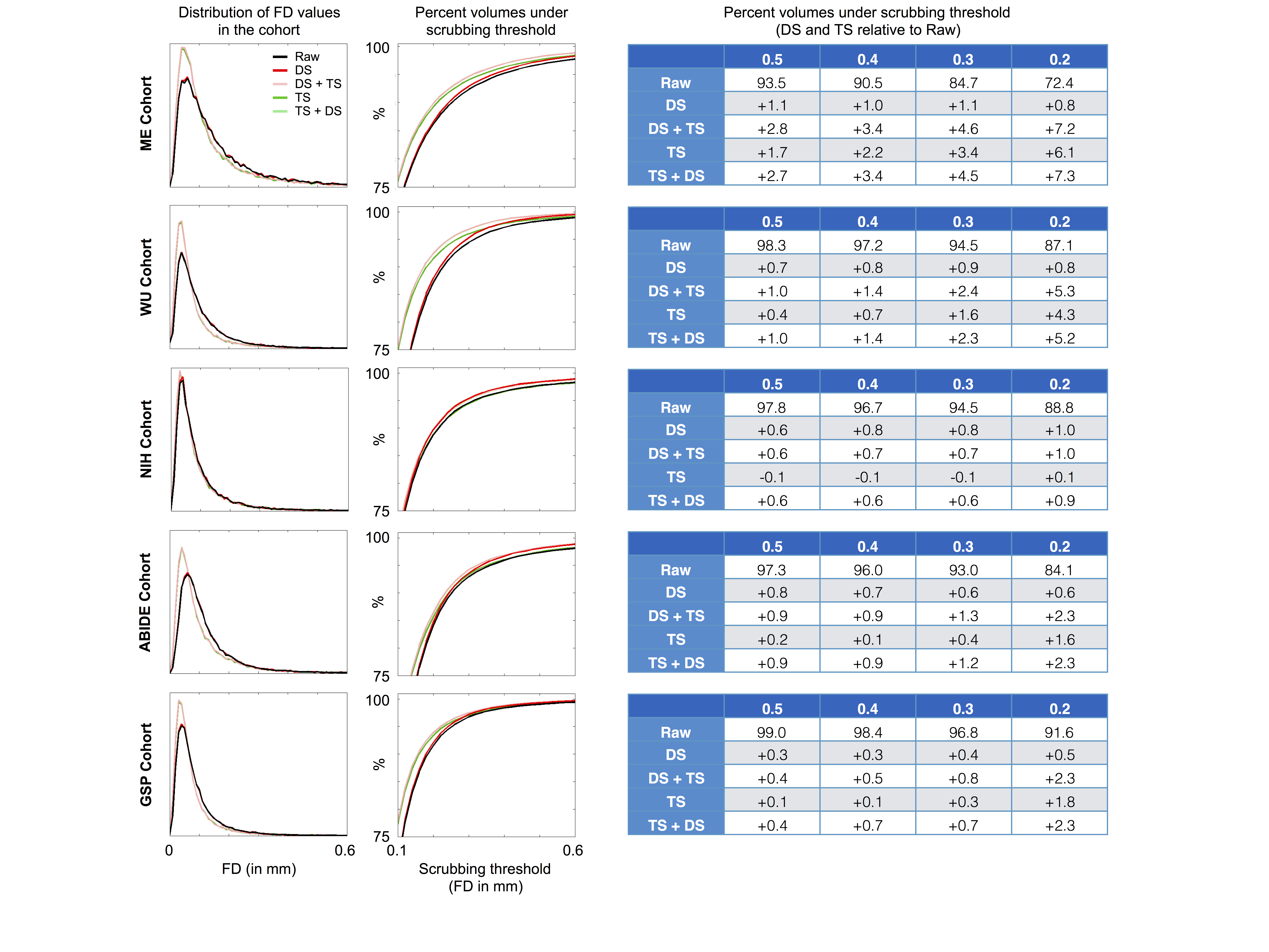

Supplement: S4 Fig — Data from each cohort is shown in a row. At left, the distribution of FD values. At middle, the percent of volumes under censoring thresholds. Values in the tables at right are drawn from the middle plots. Raw shows the percent of volumes under the threshold in raw data, and for other processing regimes, the percent additional (+) or less (-) volumes under the threshold is shown. (TIFF) [file pone.0182939.s004.tiff]

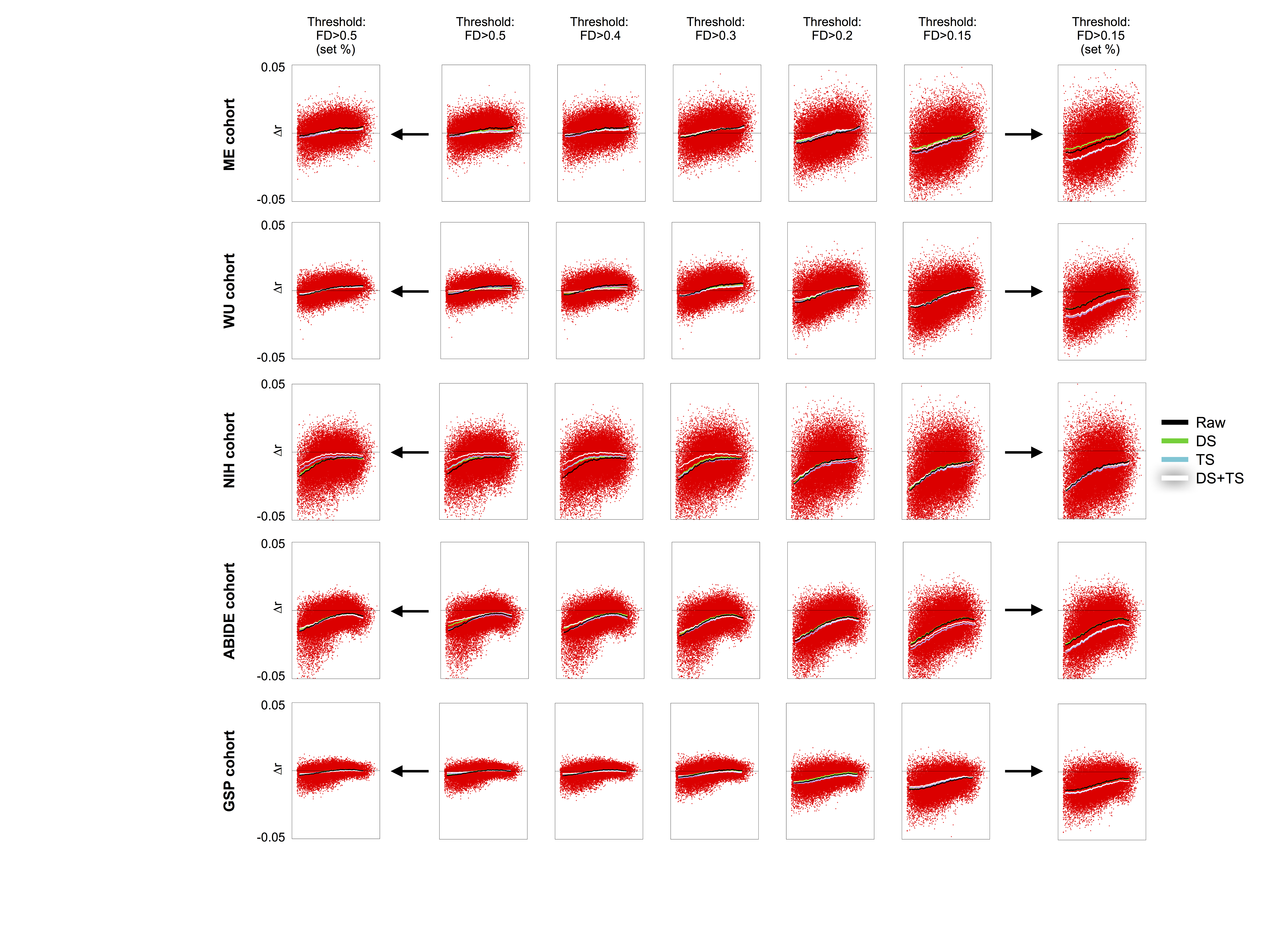

Supplement: S5 Fig — As for Fig 4 but with all cohorts. At far left and far right, rather than using a fixed threshold for all data, the percent of volumes in each subject censored by raw FD is used to define (lower) thresholds for the other post-temporal-interpolation versions of the FD traces so that all FD traces censor the same fraction of volumes. At more lenient thresholds, this has the effect of rendering post-interpolation FD traces more sensitive to motion (note that white curves better approximate the black curves). At stringent thresholds, post-slice-time correction curves (blue and white) are systematically shifted down, suggesting that they are shifted in time such that they increasingly identify a global signal that elevates correlations throughout the brain (hence the pan-distance decreases in correlations upon censoring). The x-axis represents distance, spanning 0–180 mm. (TIFF) [file pone.0182939.s005.tiff]

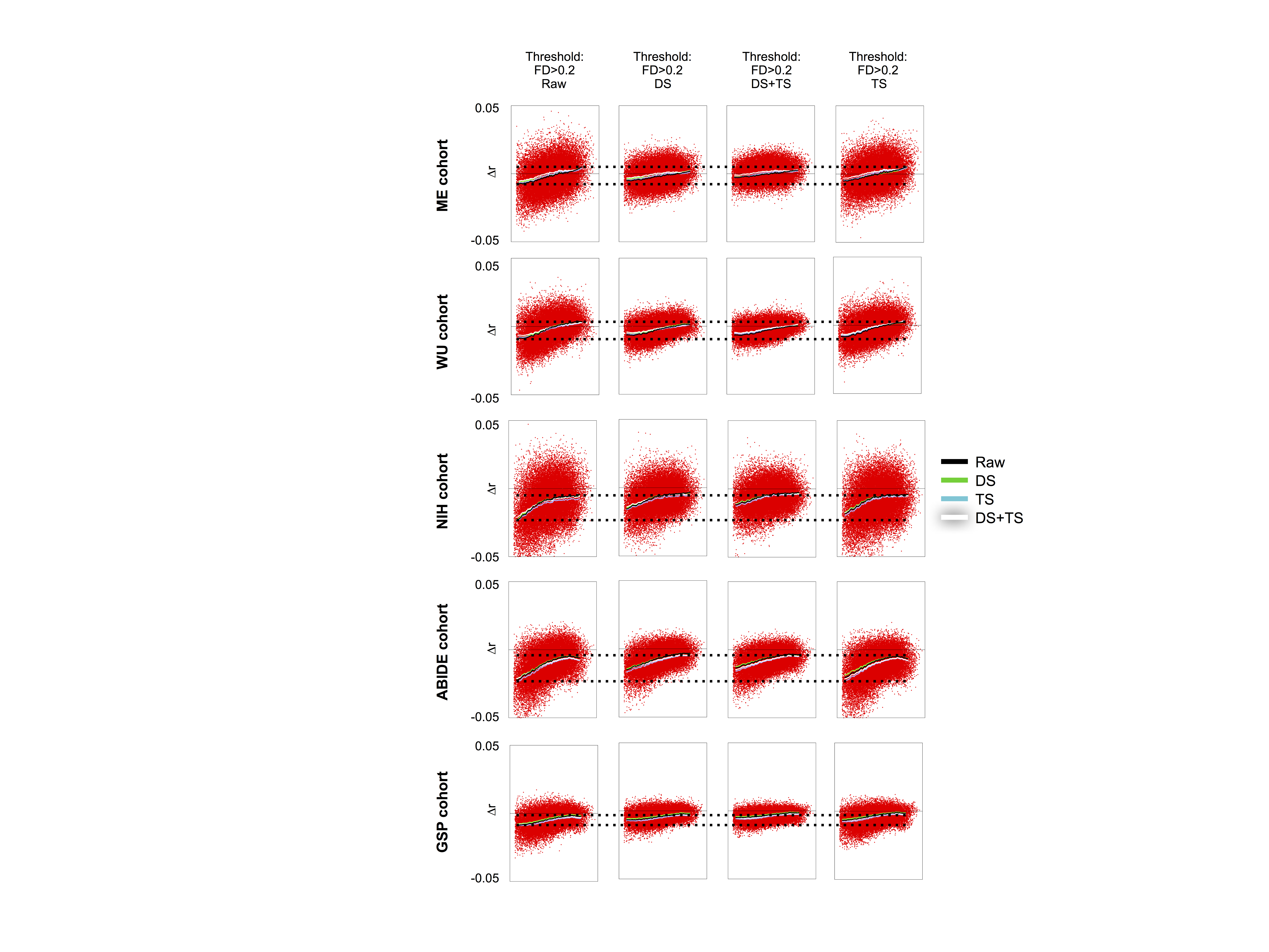

Supplement: S6 Fig — As for Fig 5, but with all cohorts. The x-axis represents distance, spanning 0–180 mm. (TIFF) [file pone.0182939.s006.tiff]

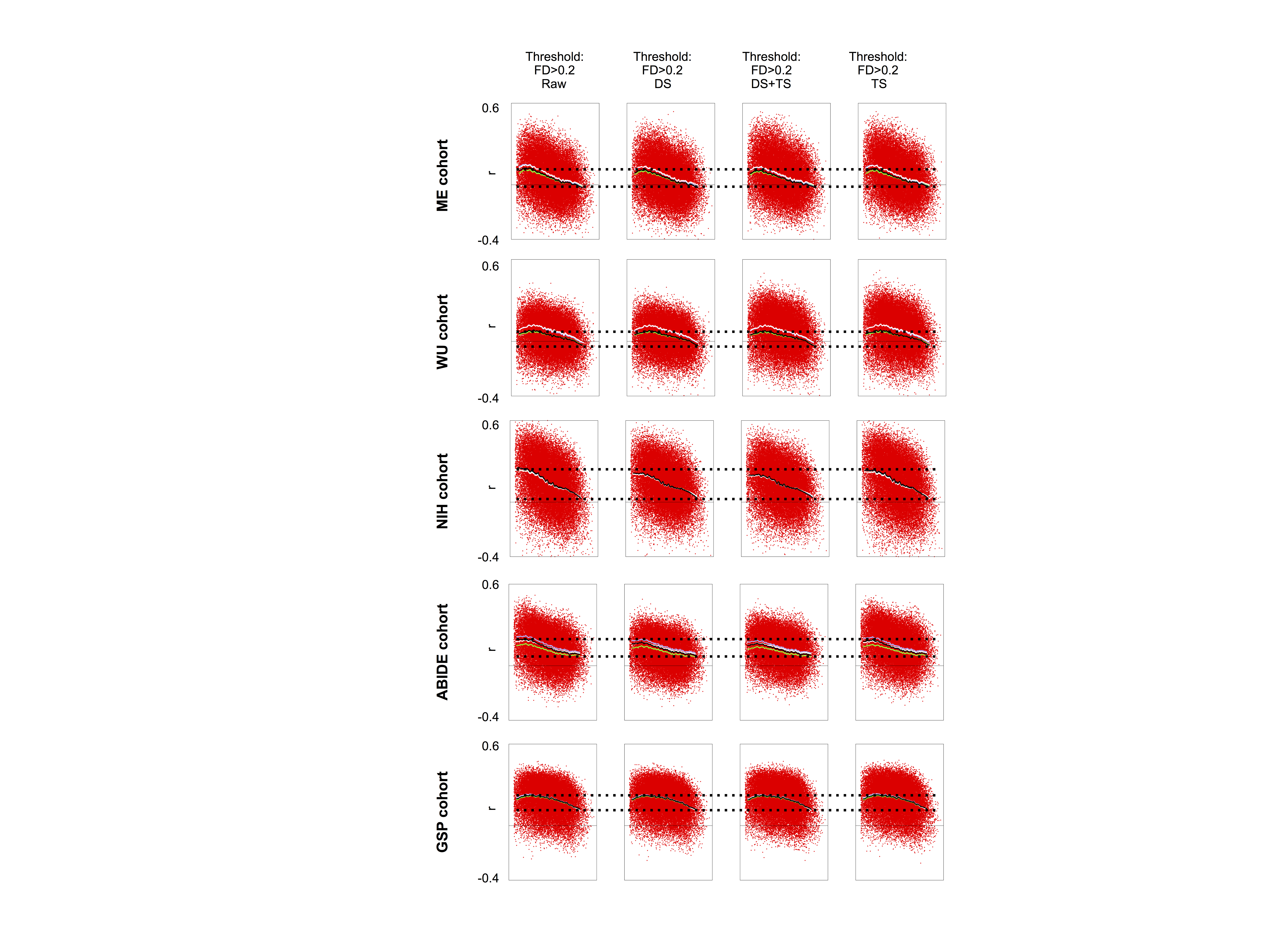

Supplement: S7 Fig — As in Fig 6, but with all cohorts. The x-axis represents distance, spanning 0–180 mm. (TIFF) [file pone.0182939.s007.tiff]
