# Supplementary material for: Temporal interpolation alters motion in fMRI scans: Magnitudes and consequences for artifact detection
Source: PLoS One. 2017 Sep 7;12(9):e0182939. doi: 10.1371/journal.pone.0182939 (PMC5589107; doi:10.1371/journal.pone.0182939)
Supplement: S1 Table — DS means despiked, TS means slice time corrected. (DOCX) [file pone.0182939.s008.docx]

| **Site** | **Average motion relative to raw estimate: mean (st. dev)** | | | | |
| --- | --- | --- | --- | --- | --- |
|  | **Raw** | **DS** | **DS+TS** | **TS** | **TS+DS** |
| **ME (N=89)** | 100 | 93 (7) | 81 (10) | 87 (11) | 81 (10) |
| **WU (N=120)** | 100 | 95 (6) | 76 (12) | 81 (13) | 76 (12) |
| **NIH (N=91)** | 100 | 92 (8) | 89 (10) | 97 (8) | 90 (10) |
| **ABIDE (N=187)** | 100 | 94 (7) | 82 (11) | 88 (11) | 82 (11) |
| **GSP (N=235)** | 100 | 97 (3) | 85 (9) | 88 (10) | 85 (9) |

Table S1: Mean and standard deviations in each dataset of the mean motion of all subjects. DS means despiked, TS means slice time corrected.
